# Preparation and Application of Thin‐Sodium Metal

**DOI:** 10.1002/smsc.202300362

**Published:** 2024-03-19

**Authors:** Shaozhen Huang, Zhiyuan He, Canglong Li, Wen Pan, Wenhao Li, Wen Liu, Tianbao Li, Yuejiao Chen, Libao Chen

**Affiliations:** ^1^ State Key Laboratory of Powder Metallurgy Central South University Changsha 410083 P. R. China; ^2^ School of Materials and Metallurgy Inner Mongolia University of Science and Technology Baotou 014010 China

**Keywords:** alloying, composite structures, interface designs, sodium‐metal batteries, thin‐sodium metal anodes

## Abstract

With the development of energy storage technology, the new energy storage materials are more diverse. The sodium metal has the advantages of high energy density, rich resource reserves, and low costs for raw materials, becoming promising advanced energy storage materials for application. However, the low tensile strength of sodium metal makes it difficult to process deformation while its severe viscosity and low melting point affect the subsequent manufactory and application of batteries. These characteristics hinder the processing and preparation of thin‐sodium metal. The designs of composite‐supporting structure, alloying, and the interface strengthening for sodium metal can effectively overcome the difficulties in preparation of the thin sodium. In this review, the design principles of thin sodium in terms of processing and preparation, according to the physical and chemical properties of sodium metal, are discussed. Meanwhile, the key challenges and new development opportunities are addressed for the processing and preparation of the thin‐sodium metal, which is beneficial for deeply understanding the reliable fabrication and realizing the practical application of thin‐sodium metals.

## Graphical abstract

This review provides three design strategies systematically (Support structure composite, interface design and alloying) for the preparation of thin sodium metal based on the characteristics of sodium metal. The electrochemical application characteristics of each design are also explained. In addition, it provides an overview of the challenges on large‐scale processing thin sodium metal.

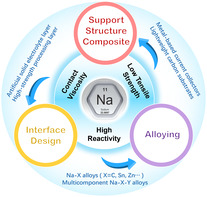

## 1 Introduction

In the past few decades, the use of traditional energy materials such as coal and oil has caused serious damage to the environment.^[^ ^1^, ^2^, ^3^, ^4^ ^]^ Thus, the demands for new energy materials are growing.^[^ ^5^, ^6^, ^7^, ^8^, ^9^, ^10^, ^11^ ^]^ In recent years, lithium‐ion batteries with the advantages of high energy density and portability have been widely used as energy storage devices in fields of 3 °C electronic products and new energy vehicles. Despite that lithium‐ion batteries can be used as one of the most promising energy storage systems, the lithium resources are scarce, making high material costs. Moreover, it is difficult to further improve the cycle life and safety of lithium‐ion batteries.^[^ ^12^, ^13^, ^14^ ^]^ Such challenges indicates that the use of lithium‐ion batteries cannot satisfy more diverse and large‐scale application scenarios.^[^ ^15^, ^16^, ^17^, ^18^, ^19^, ^20^, ^21^ ^]^

Sodium‐ion batteries have a similar industrial structure with lithium‐ion batteries, enabling them with the ability to quickly achieve a large‐scale market.^[^ ^22^, ^23^ ^]^ But the low energy density limits their further development. In contrast, sodium metal anode possesses a higher theoretical capacity, which can replace the slurry‐type graphite anode to potentially increase the energy density, making the sodium metal anode become a research hot spot in recent years. In fact, the energy density of sodium‐metal batteries (SMBs) will be affected by the thickness of sodium metal. To prepare thin‐sodium metal, researchers have studied a lot in this field to promote the application of SMBs. In practical applications, sodium metal used as an anode material is actually excessive. By thinning the sodium metal anodes, the weight and volume of the anodes can be reduced at the same time. Therefore, the battery assembled with thin‐sodium anodes has a higher energy density including volumetric energy density and mass energy density. Unfortunately, many factors hinder the preparation of thin‐sodium metal due to its physical and chemical properties.^[^ ^24^, ^25^ ^]^ Apart from low tensile strength and hardness, sodium metal owns high viscosity and low melting point, leading to poor mechanical properties of bare sodium metal to process. These bring severe challenges for the preparation of thin‐sodium metal. The high viscosity causes a lot of impurities at the interface during sodium metal processing, greatly increasing the processing difficulty. More seriously, the low melting point carries the risk of fire, which makes the process more dangerous and uncontrollable. Thus, effective techniques for the thin‐sodium fabrication are imperative for substantial developments of practical SMBs.

**Figure 1:**
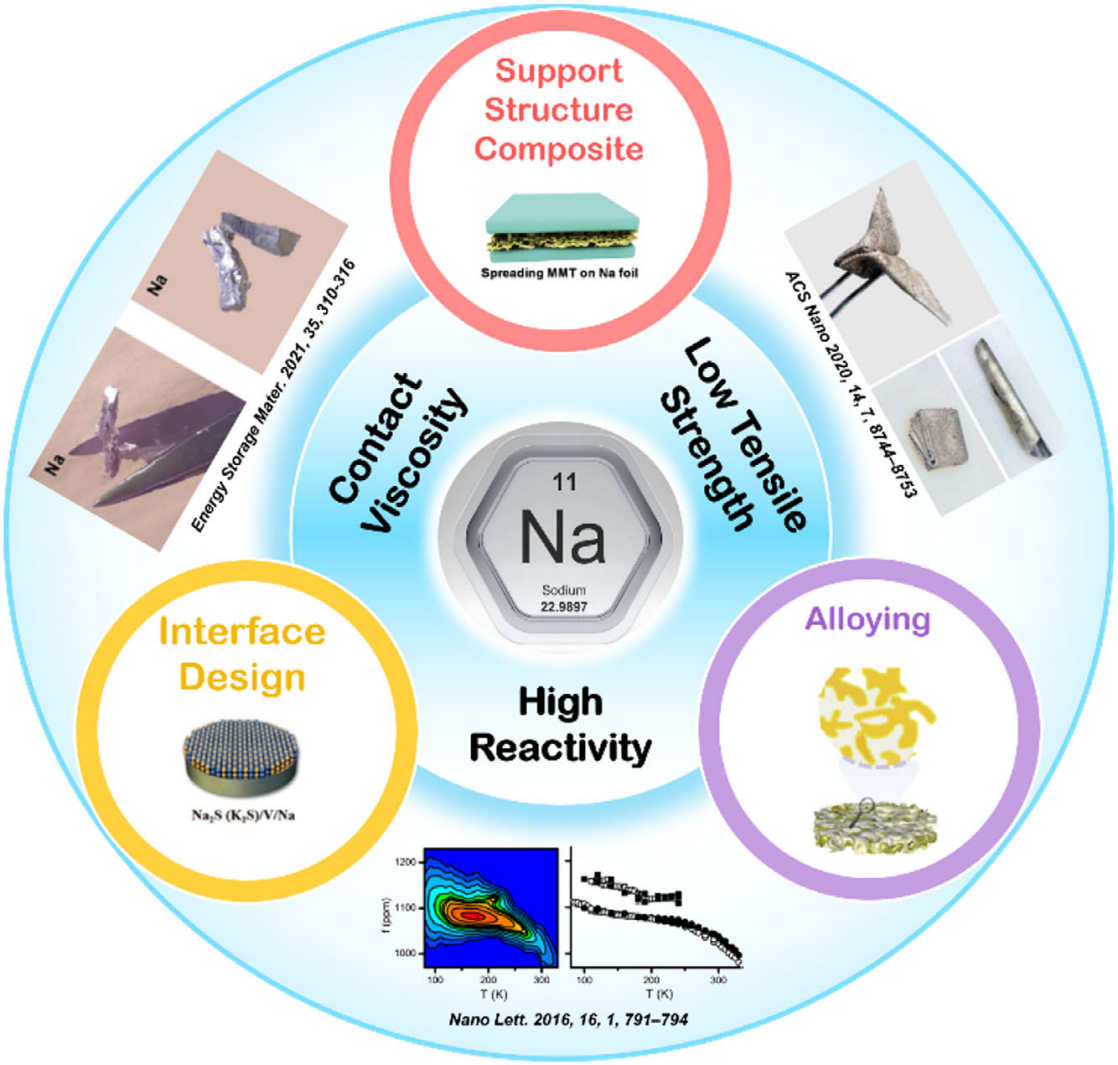
Schematic outline of the concept. Reproduced with permission.^[^ ^47^ ^]^ Copyright 2021, Elsevier. Reproduced with permission.^[^ ^63^ ^]^ Copyright 2021, American Chemical Society. Reproduced with permission.^[^ ^5^ ^]^ Copyright 2016, American Chemical Society.

As such, in this review, we discuss the design methods and the underlying principles to prepare the thin‐metal sodium, as summarized into three technical strategies shown in **Figure** **1**. The problems of low tensile strength and hardness of sodium metal can be solved by using composite‐supporting structure. Alloying the bulk phase can effectively increase the sodiophilic sites of anodes and achieve surface strengthening, thus improving the overall processing performance and electrochemical performance. In addition, designing a high‐strength interface is favor of suppressing stickiness at the interface. To solve the preparation problem of thin‐sodium metal, the current works are basically carried out the aforementioned three strategies.

Briefly, the recent research progress of thin‐sodium metals is reviewed, with emphasis on the preparation and application of thin‐sodium metals. The preparation strategies are addressed from three aspects including composite‐supporting structure, alloying, and high‐strength interface design. Some typical studies on forming thin‐sodium metal (≤200 μm) are shown in **Table** **1**. This review collects all the representative works and highlights the merits and challenges, aiming to provide a new and comprehensive knowledge for exploring thin‐sodium metal. Furthermore, the current challenges and further rational designs of thin‐Na anode are presented to bring new avenues for the development of thin‐sodium metal.

**Table 1:** Summary of thin‐sodium metal preparation methods

Synthesis design	Applications	Thickness [μm]	References
Composite‐supporting structure	Anode	50	[35]
Composite‐supporting structure	Anode	80	[36]
Composite‐supporting structure	Anode	200	[38]
Alloying	Anode	100	[45]
Alloying	Anode	100	[46]
Alloying	Anode	76.5	[48]
Interface design	Anode	100	[60]

## 2 Composite‐Supporting Structure

Constructing continuous supporting structures (such as carbon‐based belt, metal foil) can effectively assist to improve the hardness and strength of sodium metal. As a direct and shortcut method, it can successfully overcome the defects of sodium metal such as softness and viscosity to realize the preparation of thin‐sodium metal.^[^ ^26^, ^27^, ^28^, ^29^ ^]^ At the same time, due to the presence of the electrochemical deposition host brought by this method, it greatly inhibits the drastic volume change of thin‐sodium anodes during the electrochemical cycle.^[^ ^30^, ^31^, ^32^, ^33^, ^34^ ^]^

**Figure 2:**
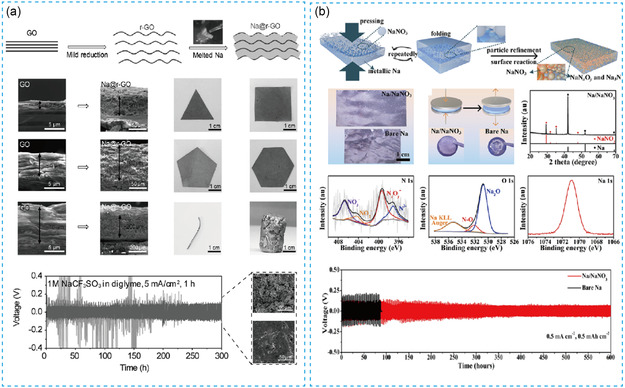
a) The scheme of preparing the thin Na@rGO composite anodes. Reproduced with permission.^[^ ^35^ ^]^ Copyright 2017, Wiley. b) Thin‐Na/NaNO_3_ composite foils were compounded by mechanical processing. Reproduced with permission.^[^ ^36^ ^]^ Copyright 2021, American Chemical Society.

**Figure 3:**
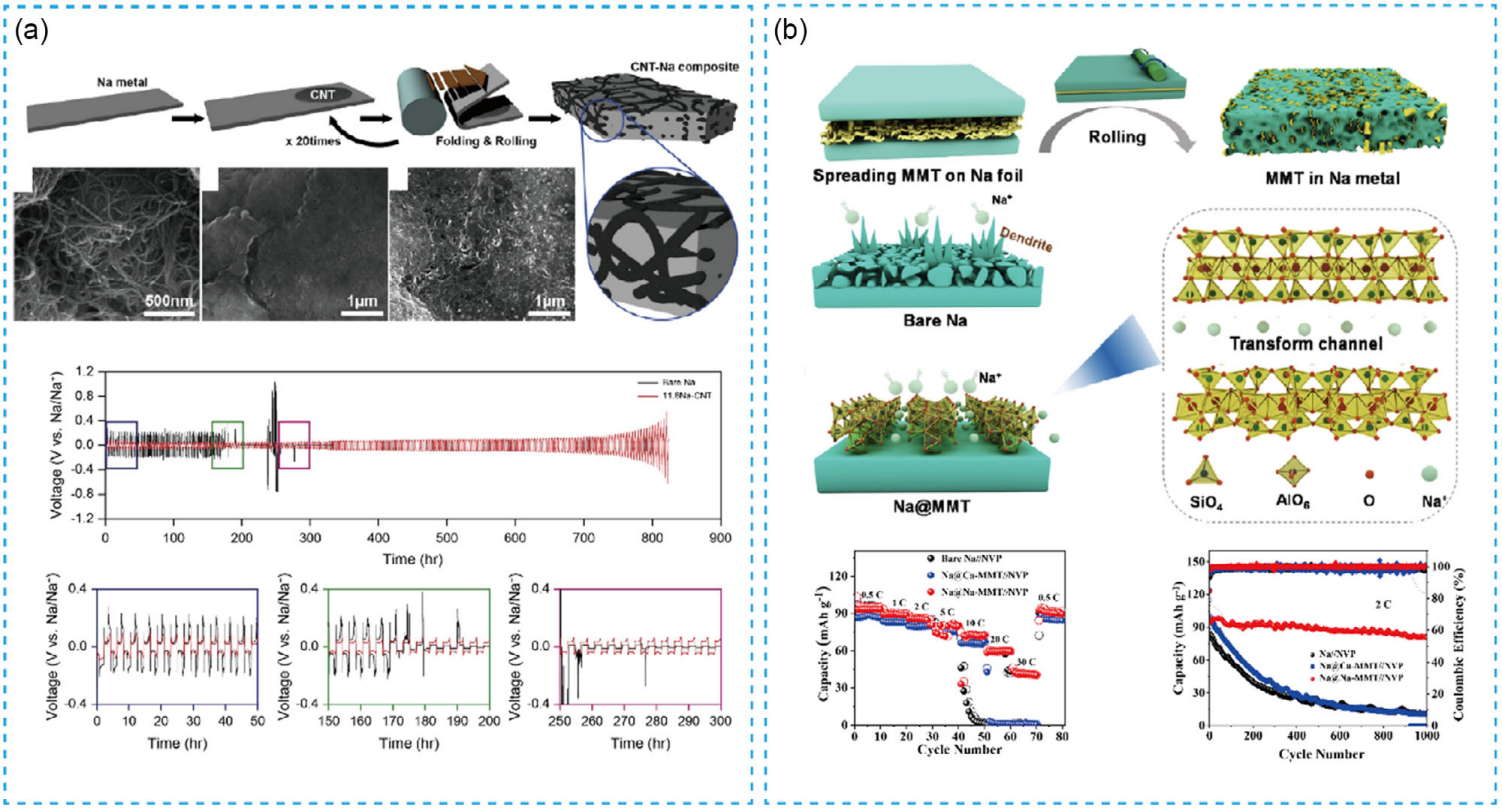
a) The negative electrode of thin CNT–Na was fabricated by crimping and folding. Reproduced with permission.^[^ ^37^ ^]^ Copyright 2019, Elsevier. b) Preparation diagram of thin‐Na@Na–MMT composite negative electrode. Reproduced with permission.^[^ ^38^ ^]^ Copyright 2022, Elsevier.

Recently, Aoxuan Wang et al.^[^ ^35^ ^]^ used molten Na and reduced graphene oxide (rGO) to prepare a thin‐sodium metal composite anode (Na@rGO) with a thickness of 50 μm, as shown in **Figure** **2**a. Compared with the pure Na metal anode, the prepared composite sodium metal with 4.5% rGO anode with a high hardness and strength can be designed in various shapes and sizes. In addition, the overall molding performance of the composite anode with good anti‐adhesion is also better and the overall strength is increased by nearly five times. The Na@rGO anodes show a stable life span more than 300 h at 5 mA cm^−2^ and 5 mA h cm^−2^ while pure sodium encounters a short circuit only after cycling for tens of hours. It proves that Na@rGO anodes can greatly improve battery stability. Xiancheng Wang et al.^[^ ^36^ ^]^ prepared thin‐sodium metals with a thickness of 80 μm by embedding NaNO_3_ in bare Na metal matrix by mechanical kneading, as shown in Figure 2b. Based on XRD and XPS results, the NaNO_3_ has existed both on the surface and in the bulk of sodium metal. The assembled symmetric cell can cycle stably for 600 h at 0.5 mA cm^−2^ and 0.5 mA h cm^−2^. Compared with the bare sodium, the mechanical processing properties of Na/NaNO_3_ composite foil are remarkably improved, accompanied by a significant decrease in the softness and viscosity. By this technology, the prepared thin anodes show better stability and conductivity, greatly improving the electrochemical performance of the battery. By a similar approach of rolling and folding, Yun‐Jung Kim et al.^[^ ^37^ ^]^ introduced carbon nanotubes (CNT) into sodium metal to prepare the CNT–Na composite electrodes with a thickness of hundreds of microns (**Figure** 1, 3), achieving more uniform Na deposition/stripping behaviors on CNT–Na anodes. The addition of a conductive CNT network in the sodium metal anode can achieve more uniform and dense Na nucleation, which is conducive to processing and improves the electrochemical performance. The symmetric cell using this thin anode displays a stable life span of 800 h at 0.5 mA cm^−2^ and 1 mAh cm^−2^, showing effective performance improvement. Additionally, Can Luo et al.^[^ ^38^ ^]^ added Na^+^–intercalated montmorillonite (NA–MMT) into sodium metal by rolling method to prepare a composite sodium metal with a thickness of ≈200 μm as a negative electrode shown in Figure 3b. Na–MMT can be homogeneously dispersed throughout the Na matrix, constructing a stable ionic conductor interface. The formed faster diffusion channels help to reduce the nuclear potential of sodium. The symmetrical cell assembled with it achieved a stable cycle of 200 h at 4 mA cm^−2^ and 4 mA h cm^−2^. The full battery (Na@Na–MMT||Na_3_V_2_(PO_4_)_3_) can realize the capacity retention up to 86% after the stable 1000 cycles with high cycle stability and excellent rate performance with discharge specific capacity 40 mA h g^−1^ at 3300 mA g^−1^. In summary, the combination of high‐strength and high‐conductivity ion‐electron support in sodium metal matrix plays a significant role in the processing and application of thin‐sodium metal anode.

Although the composite‐supporting structure can help to the processing and electrochemical application of thin‐sodium metal, there exist some problems for this method. Using the continuous support structure, it is difficult to solve the dendrite growth problem directly acted on the interface during cycling toward to the application of thin‐sodium metal at high currents densities. When the current conditions are rigor, the soild eletrolyte interphase designed by the interface can provide the mechanical force to inhibit dendrite growth directly.^[^ ^39^ ^]^ Meanwhile, the additional composite materials add the extra mass of thin‐sodium metal, decreasing the energy density of batteries assembled with the composite thin‐Na anodes. Additionally, due to the actual volume space occupied by the composite structure, the thickness of the monolithic electrode is still difficult to process thinner. Therefore, the selection of lightweight and highly conductive composite structures as much as possible is useful to improve the practical application of composite anodes.

## 3 Alloying

The Na alloying can effectively reduce the intrinsic chemical reactivity of pure sodium metal and decrease the viscosity of the interface, which is greatly beneficial for the overall processing performance of thin‐sodium metal. Furthermore, the undesirable electrochemical behaviors such as dendrites growth at low current densities and volume change during cycle can be effectively inhibited by using sodium alloy.^[^ ^40^, ^41^, ^42^, ^43^ ^]^ Also, Na alloys can suppress the side reaction between the electrolytes and the metal anode to achieve stable cycling life span, compared with the pure Na.^[^ ^7^, ^43^, ^44^ ^]^

**Figure 4:**
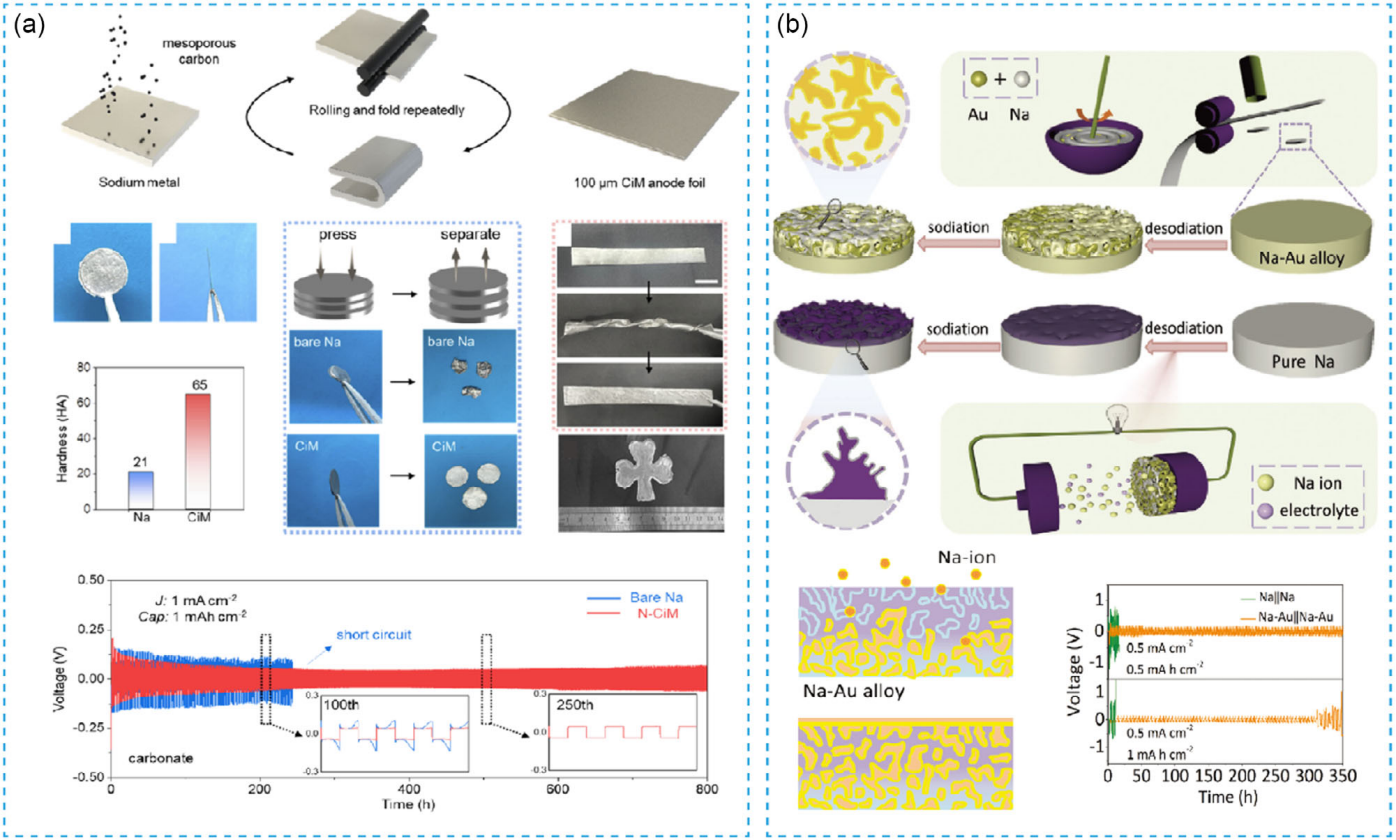
a) The process of making thin‐CiM composite foil with carbon powder and sodium metal through the tying technology. Reproduced with permission.^[^ ^45^ ^]^ Copyright 2023, American Chemical Society. b) Schematic diagram of preparing composite thin‐sodium metal negative electrode by alloy melting method. Reproduced with permission^[^ ^46^ ^]^. Copyright 2021, Wiley.

**Figure 5:**
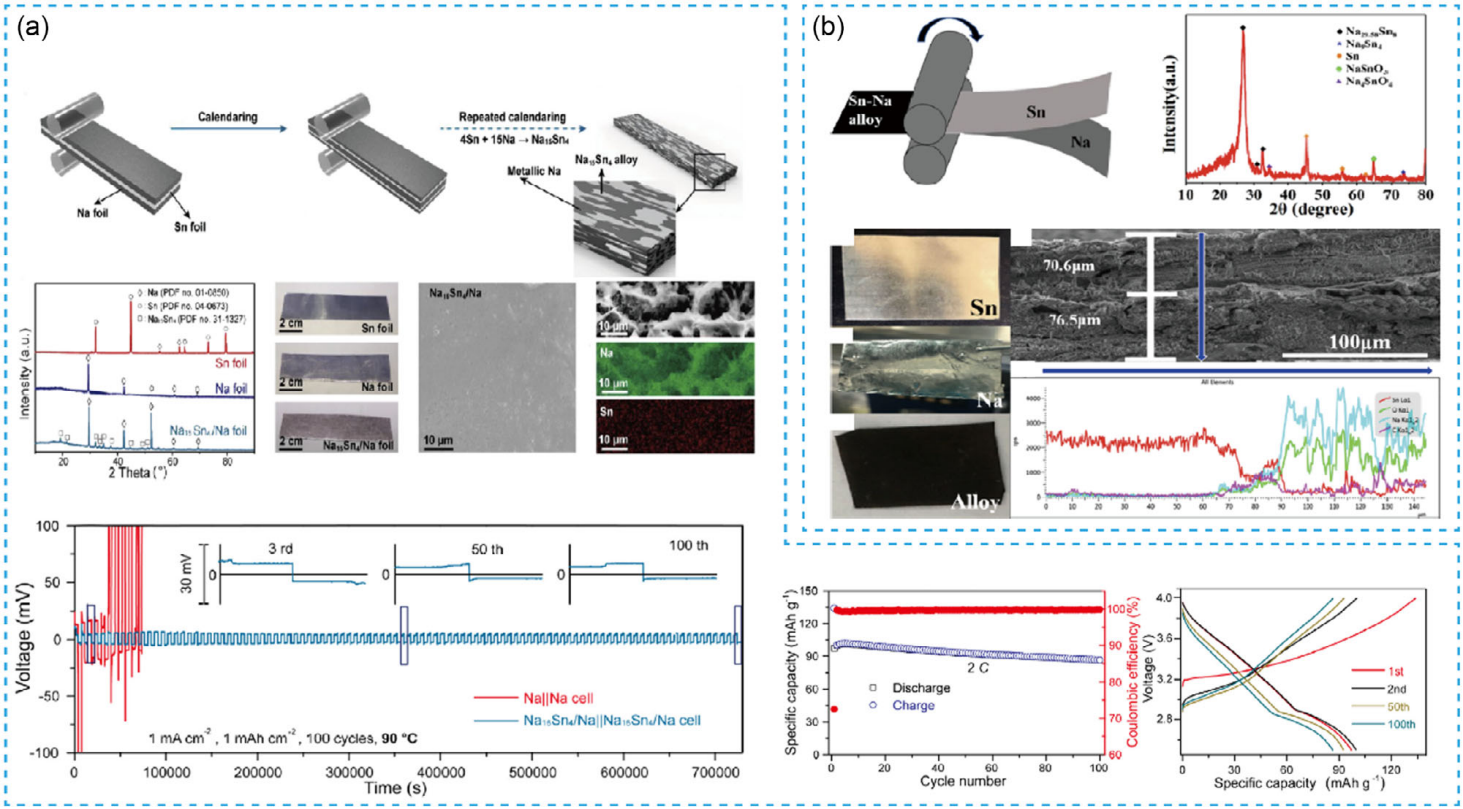
a) Diagram of preparation of thin‐sodium metal foil by cold forging technology. Reproduced with permission.^[^ ^47^ ^]^ Copyright 2020, Elsevier. b) Diagram on preparation of thin‐sodium–tin alloy. Reproduced with permission.^[^ ^48^ ^]^ Copyright 2019, American Chemical Society.

In recent works, Liu et al.^[^ ^45^ ^]^ used the mesoporous carbon (MPC) powder and sodium metal to prepare the sodium alloy anodes with a thickness of 100 μm by rolling technology shown in **Figure** **4**a, which can effectively reduce the viscosity of sodium metal and improve the strength and hardness. Due to the introduction of second‐phase carbon particles into the sodium metal, the obtained “carbon in metal” (CiM) anode has a higher hardness (65 HA) compared to bare Na metal (21 HA). Nitrogen‐doped MPC has high sodiophilicity, which can effectively promote ion diffusion and induce uniform deposition of sodium metal. Under the condition of 1 mA cm^−2^ and 1 mA h cm^−2^, the symmetric cell assembled with Na alloy electrodes stably cycle for 800 h. Furtherly, it achieves more uniform Na^+^ deposition behavior when adding nitrogen (N‐CiM) into the toner. Subsequently, the assembled cell can realize a stable life over 1200 h. The obtained full battery maintains the capacity retention rate of 88.6% after 1000 cycles. Similarly, Jiatong Han et al.^[^ ^46^ ^]^ prepared a composite sodium metal anode using alloy melting by adding Au, Sn, or In components shown in Figure 4b. This work constructed a porous structure that could not only inhibit volume change, but also increase the sodiophilic sites of the negative electrode. It showed excellent processing properties and prepared ≈100 μm thickness sodium metal anodes. To a certain extent, it is able to achieve a large capacity electrochemical cycling. The Na–Au alloy showed the best performance, with a stable cycling life span of more than 350 h at 0.5 mA cm^−2^ and 1 mA h cm^−2^. Typically, Li et al.^[^ ^47^ ^]^ prepared Na–Sn alloy metal foils with a thickness of 100 μm by cold forging technology shown in **Figure** **5**a. The XRD results indicate that the rolling method can achieve the formation of Na_15_Sn_4_. The Na_15_Sn_4_ skeleton in the alloy anode is porous from the scanning electron microscope (SEM) images. The prepared sodiophilic alloy framework can effectively reduce the current density, suppress the volume change, and achieve excellent electrochemical performance. The obtained Na_15_Sn_4_/Na anodes were assembled into symmetrical cells, showing a stable life over 200 h at 90 °C at 1 mA cm^−2^ and 1 mA h cm^−2^, with the low polarization voltage of less than 15 mV. The Na_0.9_(Cu_0.22_Fe_0.30_Mn_0.48_)O_2_||Na_15_Sn_4_/Na full battery achieved a stable cycling life span over 100 cycles at 300 mA g^−1^ with the capacity retention rate of 88%. Comparatively, the capacity retention rate assembled with pure sodium was remained only 80% under the same conditions. What's more, Wenjian Liu et al.^[^ ^48^ ^]^ were able to prepare a thin‐sodium metal of ≈76.5 μm by alloying strategy shown in Figure 5b. They take a facile roll‐to‐roll metallurgical method to prepare the Sn−Na alloy strips. This method can prepare thin‐sodium metal anodes while significantly inhibiting the gas production of the electrodes to improve the electrochemical performance of the electrodes. With the function of sodium compensation, the first Coulombic efficiency of full cell with Na_3_V_2_(PO_4_)_2_F_3_ (sodium vanadium fluorophosphate [NVPF]) cathode was increased from 24.68% (bare Sn anode) to 75% (Sn−Na alloy anode). The potential of thin‐sodium metal for pre‐sodiation in sodium‐ion batteries was demonstrated. Thinner Sn–Na metal negative electrode can be made by alloying method. In a word, the alloying strategy can improve the unfavorable processability of sodium metal and reduce the corrosion between the metal anode and the electrolytes. Furtherly, it can thinner the anodes and ensure the performance for electrochemical applications.

It is worth mentioning that the addition of too many alloying elements and amounts will reduce the content of active sodium. The addition of too few alloying elements and amounts makes the resulting alloying phase discontinuous, and the effect of suppressing volume change is limited. At the same time, the interfacial dendrite problem is not directly suppressed so that the alloy anode is difficult to apply at high currents densities. It's a middle‐of‐the‐road solution to prepare the high performance of thin‐sodium metal. Therefore, the alloy layer with the dual structure of bulk phase and interface can be considered when designing the alloyed anode.

## 4 Interface Design

In the process of preparing thin‐sodium strips, the interface is also an important aspect that needs to be noted. How to effectively overcome the contact viscosity caused by low melting point of sodium metal is an important prerequisite for processing the thin‐sodium metal. Focusing on overcoming the viscosity issue of sodium metal, many scholars have found that the design of high‐strength and heterogeneous interfaces by interface modification can improve the overall strength of sodium metal to a certain extent.^[^ ^49^, ^50^, ^51^ ^]^ From the perspective of interface design, it can also improve the processing performance of sodium metal and realize the preparation of thin‐sodium metal.^[^ ^52^, ^53^, ^54^ ^]^ Furthermore, in view of the application scenarios of thin‐sodium metal, how to design the multifunctional interface is worthy to focus on in the future research.^[^ ^39^, ^55^, ^56^, ^57^, ^58^, ^59^ ^]^

**Figure 6:**
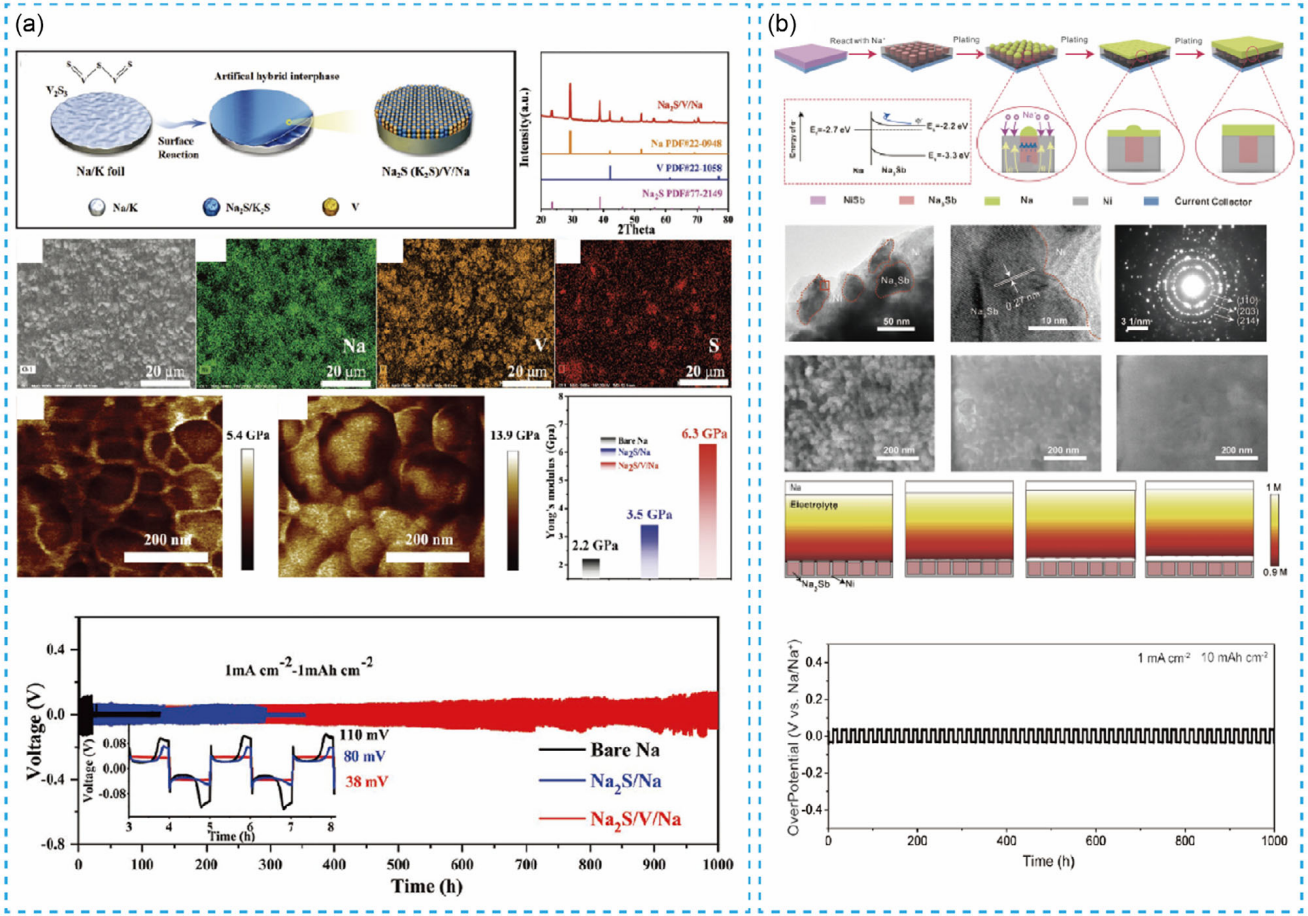
a) The diagram of preparing the thin Na_2_S. Reproduced with permission.^[^ ^61^ ^]^ Copyright 2022, Wiley. b) Schematic diagram of preparing thin‐N_3_Sb‐/Ni heterostructures. Reproduced with permission^[^ ^9^ ^]^. Copyright 2021, Wiley.

To solve the problem of electrochemical instability of sodium metal anodes, Liu et al.^[^ ^60^ ^]^ prepared 100 μm thickness thin‐sodium metal as negative electrodes with tin–sulfur/graphene nanocomposite film (A–SNS–G) by rolling technology. The interface design enables rapid ion transport and inhibits the dendrite growth. The SNS–G@Na anodes achieved stable cycle for 500 h under the conditions of 4 mA h cm^−2^ and 4 mA cm^−2^. The assembled SNS–G@Na||Na_3_V_2_(PO_4_)_3_ (NVP) full battery achieved a Coulombic efficiency of 99.69% during 600 cycles. Though this strategy displayed improved cycling stability, sodium dendrites caused by long‐term plating/stripping process will easily destroy the solid electrolyte interlayer, resulting in the severe side reaction. Thus, Jiang et al.^[^ ^61^ ^]^ coated vanadium nanoparticles and sodium sulfide (or potassium sulfide) on the surface of Na foil to form an artificial heterogeneous layer (i.e., Na_2_S/V/Na or K_2_S/V/K), as shown in **Figure** **6**a. There is a homogeneous modified layer at the interface from the SEM–electron‐dispersive spectroscopy images. The Na_2_S protective layer can enhance the strength of sodium metal, inhibit the growth of dendrites, and improve the stability of the interface. The constructed protective layer has a Young's modulus of up to ≈6.3 GPa, while the pure sodium metal is only ≈2.2 GPa. The symmetric cell assembled with the modified sodium metal (Na_2_S/V/Na) owing several hundred microns, which reveals a stably life span in the carbonate electrolytes for more than 1000 h at 1.0 mA cm^−2^ and 1.0 mA h cm^−2^. The full batteries (Na_3_V_2_(PO_4_)_3_||Na_2_S/V/Na) have a higher energy density and power density. Then, Wang et al.^[^ ^9^ ^]^ prepared an alloy interface with nickel–antimony (NiSb) (Figure 6b) to solve the problem of dendrite growth, enabling the smooth deposition on hundreds of microns of Na metal anode for effective inhibition of Na dendrites. The Na_3_Sb island is embedded in the Ni matrix at the interface shown in transmission electron microscope (TEM) images and can uniformly induce sodium metal deposition confirmed by the COMSOL simulation. This anode realized the stable cycling performance than bare Na metal for more than 1000 h under the condition of 1 mA cm^−2^ and 10 mA h cm^−2^. The full battery assembled with NVPF also displayed a capacity retention rate of more than 99.9% after 230 cycles. Similarly, Xia et al.^[^ ^62^ ^]^ used vanadium nitride (VN) powder to form VN interlayer on Na metal surface indicated by SEM and TEM images by rolling method, which can achieve uniform deposition on hundreds of microns of sodium negative electrodes. The symmetric battery with modified anode cycled for more than 1060 h at 0.5 mA cm^−2^ and 1 mAh cm^−2^. The capacity retention rate was maintained at 96% after 800 cycles at 5 °C rate in full cell assembled with NVP cathode and Na/VN anode. Thin‐sodium metal processing by reinforcing the interface can theoretically achieve extreme thickness. However, this requires a harsh strength and continuity of the interface.

The interface should be an integral design for the processing and electrochemical application of thin‐sodium metals. There are obvious shortcomings in inhibiting the volume change of sodium metal. As for it, the preferred strategy is to combine the interface design and matrix design of composite‐supporting structure or alloying. It can effectively inhibit dendrite growth while solving the lifting change. Taking this strategy will greatly expand the application scenarios of thin‐sodium metal and bring the material closer to practical applications.

## 5 Summary and Outlook

**Figure 7:**
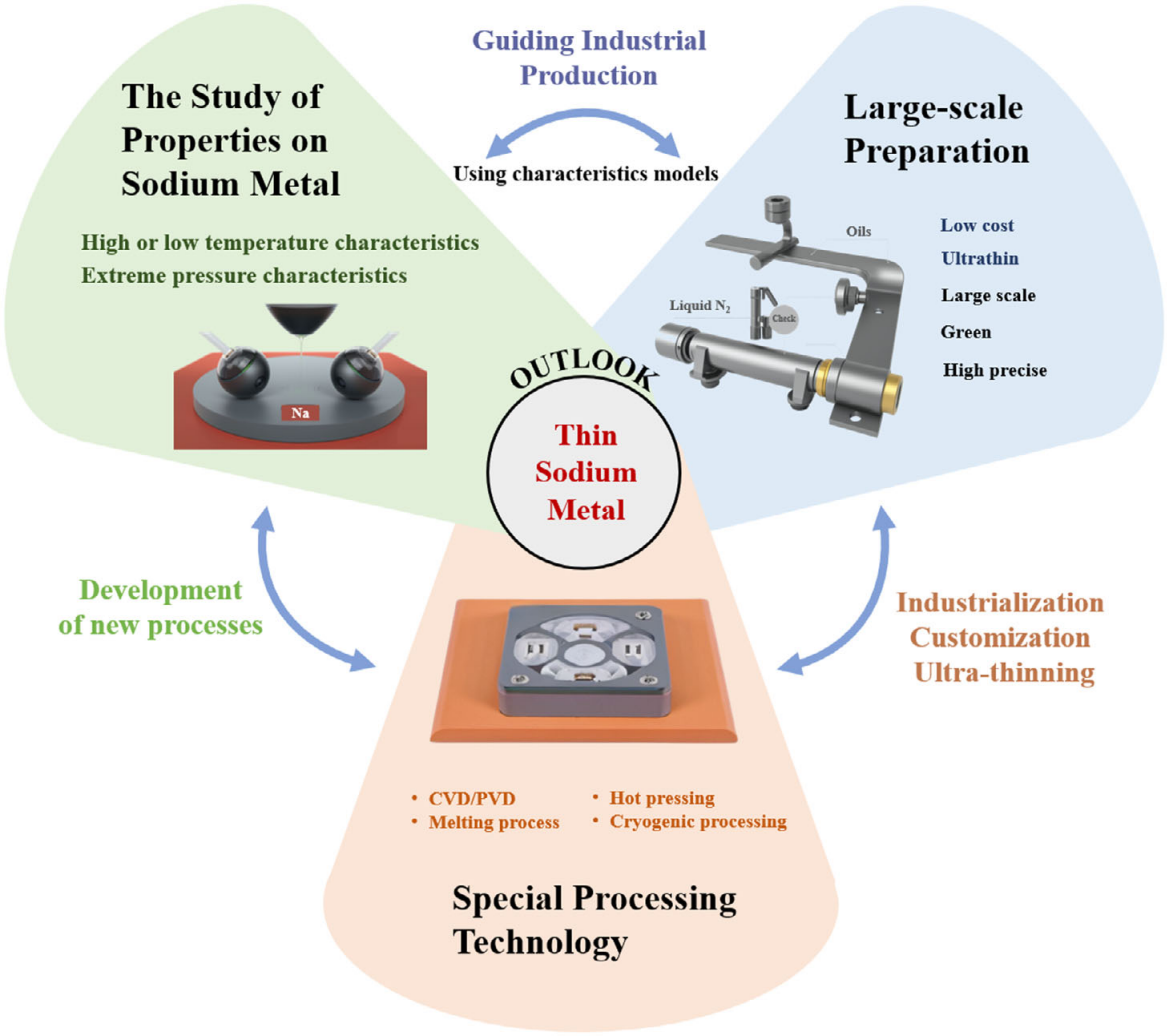
The prospect of designing large‐scale ultrathin machining technology based on in‐depth research on sodium metal properties.

With the wide application of sodium‐ion batteries and SMBs, the study of sodium metal will continue to deepen. In the design of high energy density batteries, the processing and preparation of thin‐sodium anode plays a crucial role. This article systematically reviews three design ideas for preparing thin‐sodium metal, which are based on the characteristics of sodium metal. However, the research on the preparation technology of thin‐sodium negative electrode is still at a preliminary stage, and the following aspects should be paid attention to in the future study of processing the thin‐sodium metal as shown in **Figure** **7**. 1) The research on the high‐ or low‐temperature characteristics of sodium metals should be further promoted, and there is still a scientific gap in the research field of sodium metals at the unconventional temperature; 2) The mature and large‐scale preparation environment of thin‐sodium metal still needs to be further explored to ensure the high purity and prepare the complex functional interface in the production process; and 3) For the special processing technology of thin‐sodium metal, there is still a technical gap such as the use of vacuum evaporation technology, cryogenic processing technology, and other special technology.

## Conflict of Interest

The authors declare no conflict of interest.
